# Successful Treatment of Acanthamoeba Keratitis Using 0.08% Polyhexamethylene Biguanide Monotherapy: A Report of a Case in Latin America

**DOI:** 10.7759/cureus.103935

**Published:** 2026-02-19

**Authors:** Astrid Pabon, Ivanna Agustina Lavagna, Erik Daniel Paredes Morales, Samantha Vanessa Duran Alvarez, Norka Sánchez

**Affiliations:** 1 Cornea, Centro de Investigaciones y Tratamiento Ocular, Buenos Aires, ARG; 2 Ophthalmology, Centro de Investigaciones y Tratamiento Ocular, Buenos Aires, ARG; 3 Cornea, OftalmoSalud, Lima, PER

**Keywords:** acanthamoeba keratitis, contact lens, in vivo confocal microscopy, phmb 0.08%, polyhexamethylene biguanide

## Abstract

Acanthamoeba keratitis (AK) is a rare but potentially sight-threatening corneal infection, most commonly associated with contact lens use, and frequently misdiagnosed due to its nonspecific early clinical presentation. Standard treatment regimens are prolonged and often limited by ocular surface toxicity and poor patient adherence. We report the case of a 19-year-old contact lens wearer who presented with a one-month history of ocular pain, redness, photophobia, and decreased vision in the right eye, initially treated as viral keratitis without improvement. Slit-lamp examination revealed stromal infiltrates with perineural involvement, and in vivo confocal microscopy confirmed the presence of superficial Acanthamoeba cysts without fungal elements. The patient was treated using a standardized protocol with topical polyhexamethylene biguanide (PHMB) 0.08% monotherapy, resulting in rapid clinical improvement, progressive reduction of corneal infiltrates, and complete resolution of active infection. Best-corrected visual acuity improved from 20/400 to 20/25, with no evidence of recurrence during seven months of follow-up. This case supports the potential role of PHMB 0.08% monotherapy as an effective and well-tolerated therapeutic option for AK.

## Introduction

Acanthamoeba keratitis (AK) is an uncommon but severe corneal infection that predominantly affects contact lens users and carries a high risk of visual impairment if diagnosis and treatment are delayed [1,2]. Early clinical manifestations are often nonspecific and may resemble viral, bacterial, or fungal keratitis, frequently leading to misdiagnosis and inappropriate initial therapy [3,4]. As the disease progresses, stromal involvement, perineural infiltrates, and severe inflammatory responses may develop, significantly increasing the likelihood of corneal scarring and the need for surgical intervention [5]. Perineural infiltrates are considered a highly suggestive feature of AK and reflect the neurotropic behavior of Acanthamoeba organisms [3-5].

Current first-line medical treatment for AK relies on intensive topical biguanides, such as polyhexamethylene biguanide (PHMB) or chlorhexidine at low concentrations, administered either as monotherapy or in combination with diamidines [6,7]. Although these regimens have demonstrated efficacy, they are typically associated with prolonged treatment durations, ocular surface toxicity, and challenges in maintaining patient adherence [8-10]. Moreover, Acanthamoeba's ability to form highly resistant cysts contributes to treatment failure and disease recurrence, underscoring the need for more effective and better-tolerated therapeutic strategies [11-13].

Recent clinical evidence, including data from the Orphan Drug for Acanthamoeba Keratitis (ODAK) trial and subsequent real-world case series, has suggested that higher-concentration PHMB (0.08%) monotherapy may enhance amoebicidal activity, shorten treatment duration, and improve clinical outcomes without compromising safety [13,14]. To date, most published reports and clinical trials supporting the use of high-dose PHMB (0.08%) for the treatment of AK have originated from Europe and North America, with limited representation from other geographic regions [6,7,14-16]. As a result, real-world clinical data from Latin America remain scarce. In this report, we describe a case of AK successfully treated using a standardized protocol with topical PHMB 0.08% monotherapy.

## Case presentation

A 19-year-old male contact lens wearer with no relevant systemic medical history presented to the ophthalmology service with a one-month history of progressive ocular pain, redness, photophobia, and decreased vision in the right eye (RE). The patient reported wearing monthly soft contact lenses for myopia correction and acknowledged suboptimal lens hygiene practices. He had initially been treated at another institution for presumed viral conjunctivitis with topical corticosteroids and antibiotics, without clinical improvement.

**Figure 1:**
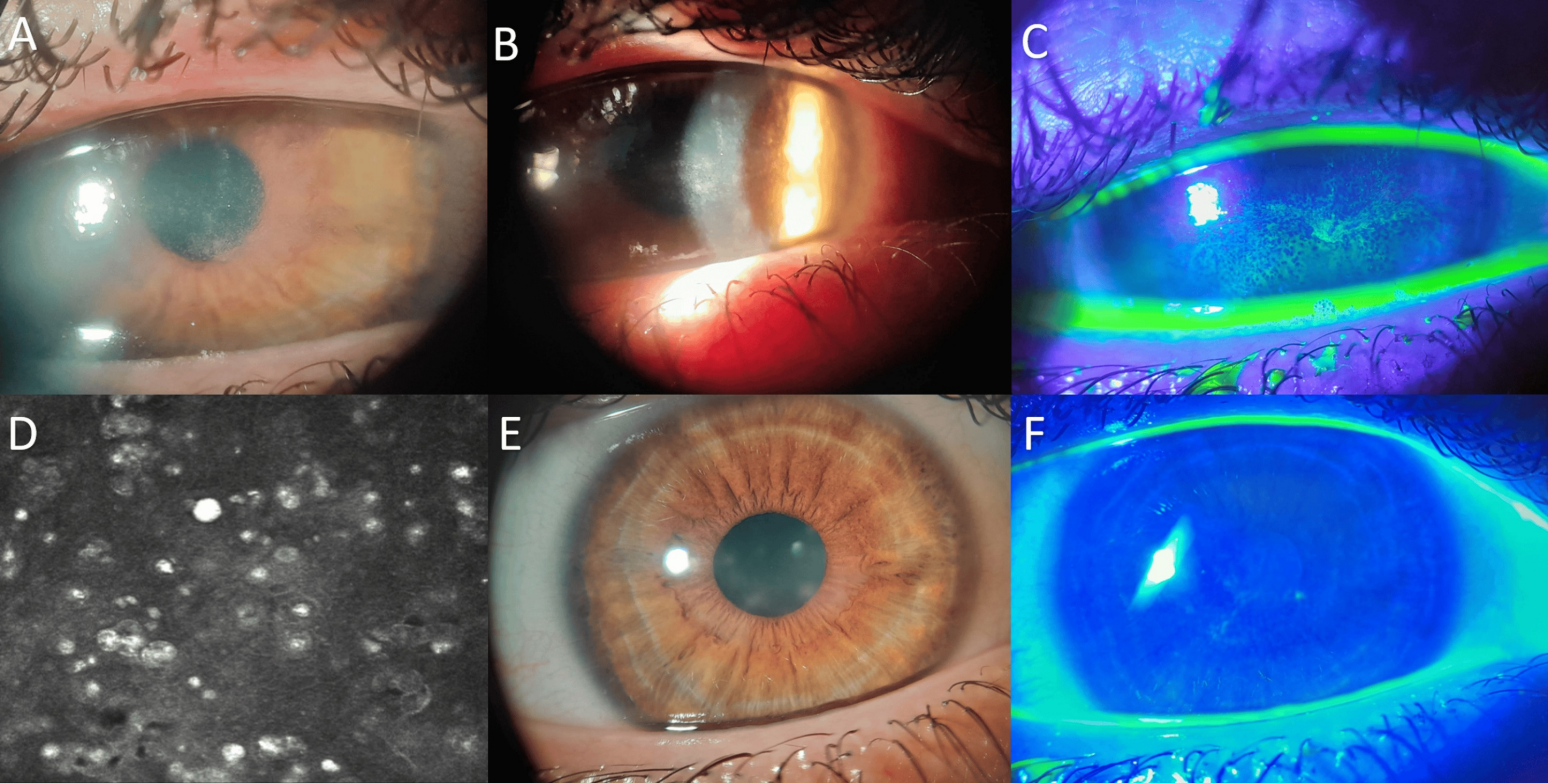
Clinical and confocal findings in Acanthamoeba keratitis before and after treatment with PHMB 0.08%. (A) Slit-lamp photograph at presentation showing central corneal opacity with stromal infiltrates and reduced corneal transparency. (B) Slit-lamp examination under oblique illumination demonstrating stromal infiltrates with a ring-shaped configuration, along with diffuse epithelial keratitis involving the pupillary axis and irregular, stellate corneal edema. (C) Fluorescein staining at presentation revealing diffuse punctate epithelial defects and positive staining over the involved corneal area. (D) In vivo confocal microscopy image showing multiple hyperreflective, double-walled structures consistent with superficial Acanthamoeba cysts. (E) Slit-lamp photograph after completion of treatment demonstrating significant clinical improvement with residual mild central stromal haze and restored corneal clarity. (F) Fluorescein staining at final follow-up showing an intact corneal epithelium with absence of epithelial defects. PHMB, polyhexamethylene biguanide.

On initial examination, best-corrected visual acuity (BCVA) was 20/400 in the RE and 20/25 in the left eye (LE). Intraocular pressure measured by applanation tonometry was 14 mmHg in the RE and 12 mmHg in the LE. Slit-lamp biomicroscopy of the RE revealed diffuse epithelial keratitis involving the pupillary axis, central corneal edema with an irregular, stellate appearance, stromal infiltrates that stained positively with fluorescein, and circumlimbal ciliary injection (Figure 1A, 1B, 1C). The LE examination was unremarkable.

Given the clinical suspicion of AK, in vivo confocal microscopy (IVCM) was performed, demonstrating superficial Acanthamoeba cysts consistent with free-living amoebae, without evidence of fungal filaments (Figure 1D). Based on these findings, a diagnosis of AK was established. Corneal scraping for culture or polymerase chain reaction (PCR) was not performed, as the diagnosis was supported by characteristic double-walled cysts identified on IVCM in a compatible clinical context. IVCM is a noninvasive diagnostic modality with high sensitivity and specificity for AK, allowing immediate visualization of cyst morphology and avoiding the potential risks associated with corneal scraping. In this scenario, additional microbiological testing was not pursued because it was unlikely to alter the initial therapeutic approach and could have delayed treatment initiation.

Treatment was initiated using a standardized protocol with topical PHMB 0.08% administered hourly for five days, with close clinical follow-up. At the 48-hour evaluation, clinical stability was observed, and the initial treatment regimen was maintained. Weekly follow-up visits were scheduled during the first month of therapy. Adjunctive topical anti-inflammatory therapy and ketorolac 0.4% eye drops were prescribed for pain control and to limit excessive inflammatory response.

After one week, the dosing frequency was reduced to every 2 hours for an additional seven days, followed by administration every 3 hours for another week. At one month, repeated IVCM demonstrated a significant reduction in the amoebic load, with persistence of superficial cysts, and the same therapeutic regimen was continued. Subsequent follow-up examinations at 60 and 90 days showed progressive improvement in corneal transparency and resolution of active inflammatory signs. A final confocal microscopy revealed a subepithelial stromal scar without evidence of viable cysts or fungal elements.

At four months of treatment, BCVA in the RE improved to 20/50, with a negative fluorescein staining pattern and residual central stromal opacity without epithelial defect. Given the favorable clinical course, the treatment frequency was gradually tapered to every 6 hours until complete resolution. After a total follow-up period of seven months, the patient achieved a BCVA of 20/25 in the affected eye (Figure 1E, 1F), with no signs of disease recurrence, and was discharged with instructions for continued periodic follow-up.

A structured summary of the clinical course is as follows: at baseline, BCVA in the RE was 20/400, with stromal infiltrates, ring-shaped lesion, epithelial defects, and superficial cysts identified on IVCM. After one month of treatment, slit-lamp findings showed reduced stromal inflammation and decreased cyst load on IVCM. At three months, BCVA improved to 20/50, with marked corneal transparency and no viable cysts on confocal microscopy. At the final seven-month follow-up, BCVA reached 20/25, with only mild residual stromal haze and no evidence of clinical or confocal recurrence.

## Discussion

AK remains a significant diagnostic and therapeutic challenge due to its low incidence, nonspecific early clinical presentation, and ability to progress rapidly to sight-threatening corneal disease [17]. Delayed diagnosis is common, particularly in contact lens users initially treated for presumed viral or bacterial keratitis [18], as occurred in the present case. Misdiagnosis and the use of topical corticosteroids in the early stages may further facilitate disease progression by suppressing local immune responses [5,19].

Accurate and timely diagnosis is critical for optimizing outcomes in AK. Conventional diagnostic methods, including corneal scraping with culture or PCR, are limited by variable sensitivity and prolonged turnaround times. In this context, IVCM has emerged as a valuable diagnostic tool, allowing direct visualization of characteristic Acanthamoeba cysts and offering higher sensitivity in experienced hands [20-23]. In the present case, IVCM played a pivotal role in confirming the diagnosis and guiding early initiation of targeted therapy. From a practical standpoint, this case reinforces the importance of maintaining a high index of suspicion for AK in contact lens users who fail to respond to conventional antiviral or antibacterial therapy [7]. Early use of IVCM in this clinical context may facilitate prompt diagnosis and timely initiation of targeted treatment, potentially preventing deeper stromal involvement and long-term visual sequelae.

The cornerstone of medical management for AK traditionally consists of topical biguanides at low concentrations, either alone or in combination with diamidines. Although effective, these regimens are often associated with prolonged treatment courses, significant ocular surface toxicity, and difficulties in maintaining patient adherence [24-26]. Furthermore, the ability of Acanthamoeba to encyst under adverse conditions contributes to treatment resistance and disease recurrence [27,28], highlighting the need for more potent and streamlined therapeutic approaches.

Recent clinical evidence has supported the use of high-concentration PHMB 0.08% as monotherapy for AK [7,16,29]. Data from the ODAK trial and subsequent real-world case series have demonstrated high rates of medical cure, reduced treatment duration, and favorable tolerability profiles, even in cases with stromal involvement [14]. The successful outcome observed in this case, characterized by rapid clinical improvement, progressive reduction of amoebic load on confocal microscopy, and complete resolution without recurrence, is consistent with these findings.

In addition, the simplified dosing strategy associated with high-dose PHMB monotherapy may represent a practical advantage over traditional combination regimens, potentially improving patient adherence during the prolonged treatment courses typically required for AK [7]. Despite the favorable outcome observed in this case, several limitations must be acknowledged, including the single-patient design and the absence of a control group, which preclude generalization of the results. In addition, the absence of microbiological confirmation by culture or PCR represents a limitation. Although IVCM is considered a sensitive and valuable diagnostic modality, microbiological confirmation remains the reference standard and would have further strengthened diagnostic certainty. It is important to note that, although favorable outcomes with high-dose PHMB (0.08%) monotherapy have been reported in the literature, most available clinical data originate from Europe and North America. Consequently, published clinical experience from Latin America remains limited, underscoring the importance of disseminating this therapeutic approach in regions where it is not yet widely incorporated into routine clinical practice. Further prospective studies and multicenter case series are needed to better define the role of this treatment strategy across different healthcare settings.

## Conclusions

AK remains a vision-threatening condition that requires early diagnosis and prompt initiation of effective therapy to optimize visual outcomes. This case demonstrates that a standardized protocol using high-concentration PHMB 0.08% monotherapy can lead to rapid clinical improvement, complete resolution of active infection, and favorable visual recovery without recurrence. While larger studies are needed to confirm these findings, this report adds real-world clinical evidence supporting PHMB 0.08% as a well-tolerated and potentially effective therapeutic option for AK, particularly in regions where clinical experience remains limited.
